# COMPARATIVE ANALYSIS OF ANTHROPOMETRIC MEASUREMENTS OF CHILDREN IN UNDERSERVED COMMUNITIES OF GUATEMALA AND DOMINICAN REPUBLIC

**DOI:** 10.51894/001c.123103

**Published:** 2024-08-30

**Authors:** Emma Schaefer

**Affiliations:** 1 College of Osteopathic Medicine Michigan State University

## 86

### BACKGROUND

From 1997 to 2003, the World Health Organization (WHO) created the Multicentre Growth Reference Study (MGRS) to establish international standardized growth charts for infants and young children. The study included data of 8,500 children from six countries. We took anthropometric measurements in the Dominican Republic and Guatemala to assess the WHO standards.    

### OBJECTIVE

The MGRS includes only one Latin American country. Our hypothesis is that the MGRS would not accurately represent the growth of children in Latin America. We argue that each region and country should develop their own charts. 

### METHODS

Children (3 months to 5 years of age) from Guatemala and the Dominican Republic were measured in accordance with WHO guidelines after guardian consent. Information including community of origin, dwelling type, school attendance, parental education level, household income, breast feeding, and disabilities was collected from the guardian. Medical Students (supervised by physicians) performed measurements including weight, height, head circumference, triceps and subscapular skinfolds, and BMI. Z-units using WHO Anthro software were used to compare our data to WHO growth charts. Bland-Altman plots showed the degree of agreement between expected and observed values.    

### RESULTS

Measurements of Guatemalan children show slight variation from ranges demonstrated by the WHO guidelines. Guatemalan children heights and weights were below the WHO guidelines but were not statistically significant. Despite Guatemala and Dominican children living in the same Caribbean region, there was a significant difference between weight for age and height for age. Guatemalan children were significantly shorter (t = -4.660, p<0.001) and weighed less (t = -3.101, p=0.002) than Dominican children. Bland-Altman plots suggest a weight difference between the Guatemalan and Dominican children of -1.05 kg between the two groups and a height difference of -2.05 cm.   

### CONCLUSION

Although both data sets were small, it suggests that the children’s measurements vary significantly from one community to another. This reinforces the idea that growth charts should be more regional for proper estimates of development. WHO charts seemed to work better for the Guatemalan children in comparison to the Dominican children, however, there was still variations that could be explained by local growth charts.

